# Research on the Decoupling of Water Resources Utilization and Agricultural Economic Development in Gansu Province from the Perspective of Water Footprint

**DOI:** 10.3390/ijerph17165758

**Published:** 2020-08-09

**Authors:** Changfeng Shi, Hang Yuan, Qinghua Pang, Yangyang Zhang

**Affiliations:** 1 Business School, Hohai University, Jinling North Road NO.200, Changzhou 213022, China; 1763510233@hhu.edu.cn (H.Y.); 20031615@hhu.edu.cn (Q.P.); 2 School of Economics and Management, Southeast University, Nanjing 211189, China; zhangyangyang0826@163.com

**Keywords:** water footprint, tapio decoupling, LMDI, driving factors

## Abstract

Objectively evaluating the decoupling status of water resources utilization and economic development is an important sign of judging the sustainability of regional economic development. From the perspective of water footprint (WF), this paper expands the scope of water resources accounting by assessing agricultural blue WF, green WF and gray WF. The Tapio decoupling index was used to explore the decoupling status of agricultural WF and economic development in Gansu Province from 2006 to 2015, and the logarithmic mean divisor index (LMDI) decomposition model was used to identify the main driving factors of agricultural WF changes and explore the degree of divergence between agricultural economic development and water resources utilization. The results showed that agricultural economic growth was a main factor for the increase of WF; the improvement of agricultural production technology had a restraining effect, and the population effect and structural effect had a lesser effect. During the research period, the relationship between agricultural WF and economic growth in Gansu Province changed from weak decoupling to strong decoupling, and the contributing factors to decoupling were in descending order: economic, technological, structural and population. Finally, this paper puts forward suggestions on optimizing planting structure, improving agricultural technology and economic development mode to promote the sustainable development of local agriculture.

## 1. Introduction

Water resources are the basic resources for the development of agricultural economy, and the shortage of water resources has become an important factor restricting Chinese agricultural development. With the continuous improvement of social and economic development, combined with the spatial and temporal differences in the distribution of water resources, the contradiction between water resources demand and agricultural development has become increasingly prominent. Therefore, studying the correlation between agricultural water resources utilization and economic development is of great significance for ensuring the normal function of regional agricultural functions and promoting regional sustainable development. Research on the relationship between economic growth and resources and environment has been a wide concern of scholars all over the world. Early scholars used a linear model to discuss the relationship between economic growth and resources and environment and then used a nonlinear model and developed the logarithmic curve model [1] and non-parametric model [2]. Grossman et al. (1995) [3] proposed an inverted “U”-shaped environmental Kuznets curve and then explored the relationship between economic and social development and natural resource environment. The Organization for Economic Cooperation and Development (OECD) [4] proposed the concept of decoupling in the sense of economics and explored the relationship between economic growth and environmental pollution through model construction. Compared with the previous method, the decoupling model can more directly reflect the degree of divergence between economic development and resource utilization. From the perspective of traditional water resources utilization, agricultural water mainly includes irrigation water, and there is a lack of accounting for the “green water” involved in the evapotranspiration of plants during the crop growth cycle. In the 1990s, English scholar Allan (1993) [5] proposed the concept of virtual water to reflect the amount of water consumed by people during production and service. Hoekstra et al. (2002) [6] proposed the concept of water footprint for the first time in combination with virtual water and ecological footprint theory. By calculating the production and domestic real water consumption, it expanded the scope of water resources accounting and overcame the problem that previous research on water use efficiency could not truly reflect water resource consumption. The concept of water footprint has gradually become one of the perspectives to study the relationship between water resources utilization and economic development.

**Figure 1:**
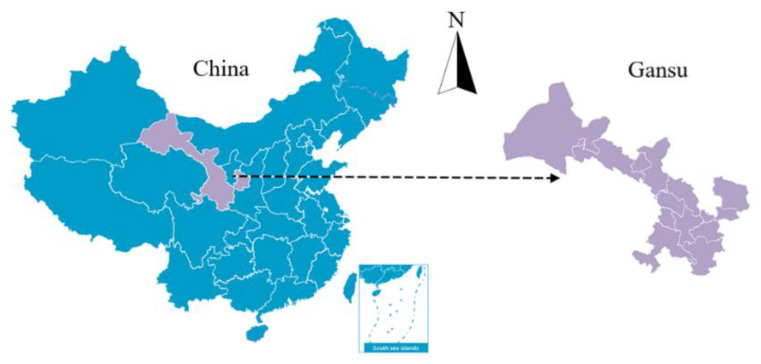
The location of the research area.

Gansu Province is located in the northwest inland of China (Figure 1), with a dry climate and lack of water resources. The multi-year average of water resources in Gansu Province is 230.6 × 10^8^ m^3^, and the per capita water resource is 888.97 m^3^, which is much lower than the national average. Furthermore, Gansu’s agricultural water consumption accounts for 79.4% of the total water. The lack of water resources severely restricts the local ecological environment and agricultural economic development. In view of this, this paper takes Gansu Province as the research object and studies the decoupling relationship between agricultural water use and economic development from the perspective of water footprint. The contribution of this paper has the following two points. The first is that based on the perspective of water footprint, by measuring the agricultural water footprint, the water footprint method is introduced into the decoupling analysis to study the decoupling relationship between water use and economic development in the agricultural sector. The second is to decompose the decoupling index of agricultural water footprint in Gansu Province, to explore the main driving factors for the decoupling of agricultural water footprint in this region, and the degree of divergence between agricultural economic development and agricultural water footprint. The paper takes into account the actual situation of crop planting structure in Gansu Province. Firstly, a bottom-up accounting method is used to quantitatively measure the blue water footprint, green and gray water footprint of staple and cash crop in production processes. Secondly, the Tapio decoupling index is used to discuss the decoupling status of agricultural water footprint and economic development. Thirdly, the logarithmic mean divisor index (LMDI) decomposition model is used to identify the main driving factors of agricultural water footprint changes and explore the degree of divergence between agricultural economic development and water resource utilization. Finally, with a view to providing practical suggestions for the sustainable agricultural development and water ecological security in Gansu Province.

## 2. Literature Review

The Tapio decoupling model has been widely used in the field of resources and environment since the decoupling theory was proposed, and the decoupling status of water resources utilization and economic development has become an important indicator for judging regional sustainable development. Qiu et al. (2018) [7] chose the improved Tapio decoupling theory and Stirpat model to explore the spatial difference and decoupling degree of economic growth and groundwater consumption along the Yellow Sea and Bohai Sea coasts. Wang et al. (2018) [8] quantified the relationship between urban economic growth and water use in Beijing, Shanghai and Guangzhou by establishing a water resource decoupling model and a water environment decoupling model. Cai et al. (2019) [9] adopted the Hodrick–Prescott filter decoupling index analysis model to study the decoupling status of water resources and economic development in the Poyang Lake Basin based on the decoupling index and spatial migration characteristics. Zhao et al. (2019) [10] applied the environmental Kuznets curve (EKC) and decoupling model and systematically studied the relationship between the water footprint and economic development of China’s textile industry from 2001 to 2012. Tian et al. (2017) [11] selected the grain output of China’s five major grain crops as research objects and used IPAT identity and decoupling analysis to assess the scale of water footprint consumption, factors affecting water footprint fluctuations. Zhang et al. (2014) [12] conducted the decoupling analysis of agricultural water consumption and environmental impact from crop production of commercial grain from 2000 to 2009, based on two indicators, DY-WC and DY-Wei. More and more studies integrate the water footprint method into the decoupling analysis, further expanding the scope of application of decoupling model.

The assessment and evaluation of water footprint is the premise and foundation of water footprint research. Chapagain et al. (2011) [13] focused on the estimation of evapotranspiration from rice fields and based on a higher spatial resolution and local data on actual irrigation, evaluated the green, blue and gray water footprint of rice from production and consumption perspectives on a global scale. Chen et al. (2017) [14] used an inter-regional input–output model to quantitatively measure the water footprint of various provinces in China, and the inter-provincial transfer of virtual water. Stella et al. (2019) [15] introduced a reduction factor in plant coefficients, measured the water footprints of all the main crops on Rhodes island and analyzed the types of water footprint components. Vale [16] et al. (2019) adopted the Paraiba model to calculate the gray water footprint of the pesticide mixture in a soil cultivated with sugarcane in Brazil. Nezamoleslami et al. (2020) [17] applied the concept of water footprint to Iran’s industrial sector based on the water footprint network method and life cycle assessment framework and proposed an improved water footprint model for steel production to assess the water footprint of personnel’s foods. Zheng et al. (2020) [18] combined water footprint and three water productivity indexes (WPI, WPU and WPET) to quantitatively study the impact of climate change on water footprint in Kaifeng and Kunshan rice production and predicted future green water changes in footprint and blue water footprint. As more and more scholars conduct research based on the water footprint perspective, water footprint assessment and evaluation methods have been continuously improved and developed as well.

The logarithmic mean divisia index (LMDI) proposed by Ang et al. (1998) [19] is widely used in the analysis of driving factors of carbon emissions and water consumption. Wu et al. (2019) [20] used the LMDI decomposition method to study the impact of energy structure, energy intensity, economic output, and population size on China’s provincial carbon emissions. Zhao et al. (2018) [21] decomposed the driving factors of agricultural carbon emissions into economic output of water resources, water-land resource ratio, population factor, per capita land use area and agricultural carbon emission intensity and discussed the relationship between water and land resource development and agricultural carbon emissions. Yao et al. (2019) [22] applied the LMDI decomposition method to the analysis of water intensity, decomposing the spatial and temporal differences in water intensity into intensity effects and structural effects. Zhang et al. (2018) [23] used the LMDI model to measure the contribution of each driving factor to agricultural water use, thereby quantitatively analyzing the main driving factors of agricultural water use at different stages in the Heihe river basin of China. Zou et al. (2018) [24] used the LMDI decomposition model to quantify the changes in irrigation water demand driving factors from 1985 to 2014 and identified four driving factors including planting scale, planting pattern, climate change and water-saving technologies.

In addition, some scholars have applied the LMDI decomposition method to the fields of water footprint and used it to decompose and study the driving factors of water footprint changes. Based on the LMDI decomposition model, Zhao et al. (2014) [25] used a bottom-up method to measure China’s agricultural water footprint and decomposed the potential driving factors for agricultural water use into dietary structure effects, efficiency effects, economic activity effects and population effects. Xu et al. (2015) [26] comprehensively considered the direct and indirect water use and used the LMDI model to quantitatively analyze the driving factors of the change in the water footprint of China’s grain crops from 1978 to 2012. Zhao et al. (2017) [27] quantitatively studied the blue and green water footprints of major crops in Suzhou, China, focusing on the driving factors related to farmland, using the LMDI model to decompose the water footprint changes from 2001 to 2010.

The LMDI decomposition method is often combined with the Tapio decoupling index, which is widely used in environmental energy area such as resource consumption. Liang et al. (2019) [28] combined the LMDI model and Tapio decoupling model to decompose the changes of carbon emissions and analyzed the dynamic evolution of carbon emissions characteristics of China’s energy-intensive industries and sub-sectors. Wang et al. (2018) [29] combined the LMDI model with the Tapio model to develop a new decoupling model for analyzing the relationship between urban industrial water use and economic development. Li et al. (2017) [30] measured the changes in the blue water footprint, gray water footprint and total water footprint of the Chinese textile industry from 2001 to 2014, used the Tapio decoupling model to study the relationship between the water footprint and economic growth and determined the factors that affected the water footprint of the textile industry with the help of LMDI. In summary, previous studies have shown that the LMDI decomposition method and the Tapio decoupling model are more applicable to the decoupling research of economic development and resource utilization and can clearly show the key indicators that affect the decomposed factors. Most of the previous studies focused on the relationship between water consumption and economic development of the whole regional economic system or industrial and textile sectors. However, there is still not much research on the agricultural sector that consumes the most water, as well as the study on the relationship between agricultural water use and economic growth. At the same time, considering that the water footprint method uses multi-dimensional indicators, which can explain the agricultural water consumed or polluted in crop production according to its elements and sources, this study integrates the water footprint (WF) method into the decoupling analysis.

## 3. Methods and Model

### 3.1. Water Footprint Evaluation

**Figure 2:**
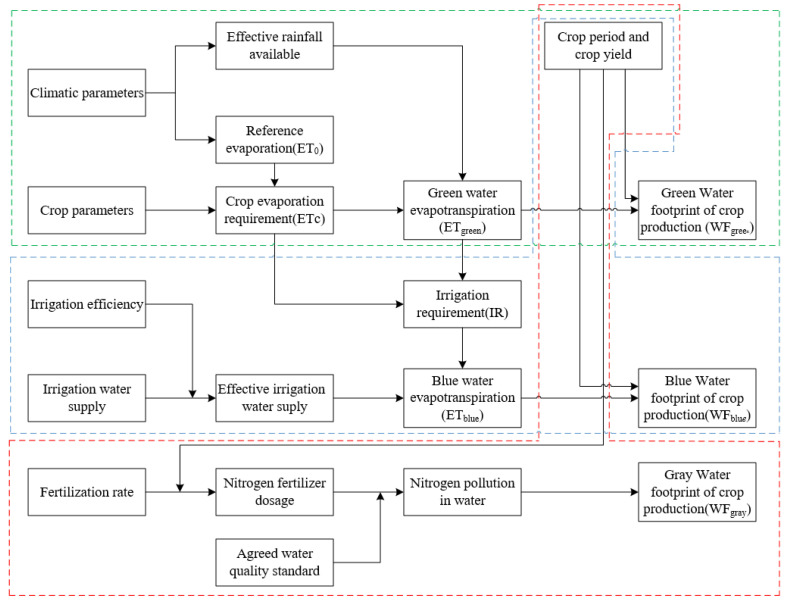
Diagram of calculation of the water footprint (WF) of crops in Gansu Province.

Hoekstra et al. (2009) [31] proposes that the water footprint includes three parts: blue WF, green WF and gray WF. In detail, the blue WF refers to the consumption of surface water and underground irrigation water. Green WF represents the evaporation of rainwater supply in crop production. Gray WF refers to the amount of fresh water required to absorb pollutant loads during the production process (2014, 2011) [32,33]. The calculation of WF of crops in Gansu Province is shown in Figure 2, the green, blue and red dashed outline, respectively, represent the calculation of green WF, blue WF and gray WF. Figure 2 is adapted from Chapagain et al. (2008) [34], Aldaya et al. (2008) [35] and Zhang et al. (2014) [12], which is added calculating of the gray WF.

A bottom-up accounting method is used to calculate the WF of crop production [36]. The WF of crop production is the sum of blue, green and gray water components [37], calculated separately for the 3 staple crops, including rice, corn and wheat; 3 cash crops, including cotton, soybeans and potatoes.

(1) $$\mathrm{WF}=\underset{i}{\sum }{\mathrm{WF}}_{i}$$

WF = WF_blu*e*_ + WF_green_ + WF_gray_ (2)

where WF is the total water footprint of local crop production in Gansu Province (m^3^ year^−1^), WFi means WF of each type of crop in Gansu Province, WF_blue_ represents blue WF (m^3^ year^−1^), WF_green_ refers to green WF (m^3^ year^−1^), and WF_gray_ is gray WF (m^3^ year^−1^).

(1) The blue WF of crop production is mainly represented by irrigation water (IR), which equals to the actual planted area (hm^2^) multiplied by the irrigation quota (m^3^ hm^−2^) per year. The irrigation quota here varies according to the type of crop and rainfall in a year, which entirely depends on the actual situation of crop planting in Gansu Province.
(2) Green water footprint here was represented by effective rainfall or crop evaporation, which can be estimated with the CROPWAT8.0 model:
  WF_green_ = 10 × ET_green_ × A (3)
  ET_green_ = min (ET_c_, P_eff_) (4)
  where ET_green_ is green water evapotranspiration (mm); that is the crop evapotranspiration during the growth period (mm). ET_c_ refers to the crop evapotranspiration (mm); P_eff_ is the effective precipitation (mm); A is the plant area of calculated crops (hm^2^); the factor 10 converts water depth (mm) into water volume per acreage (m^3^ hm^−2^). ET_c_ and P_eff_ can be directly calculated by using CROPWAT8.0 model.
  ETc was calculated by reference evaporation along with crop factors. The calculation of reference evaporation (ET_0_) was derived from the latest revised F.A.O. Penman–Monteith method, given by the following relationship [38]:
  (5) $${\mathrm{ET}}_{0}=\frac{0.408\Delta \left(R_{n}-G\right)+\gamma \frac{900}{T+273}u_{2}\left(e_{s}-e_{a}\right)}{\Delta +\gamma \left(1+0{.34u}_{2}\right)}$$
  ET_c_ = ET_0_ × K_c_ (6)
  where ET_0_ is reference evapotranspiration (mm day^−1^); R_n_ is net radiation at the crop surface (MJ m^−2^ day^−1^); G represents the soil heat flux density (MJ m^−2^ day^−1^); T refers to mean daily air temperature at 2 m height (°C); u_2_ is the wind speed at 2 m height (m s^−1^); e_s_ is the saturation vapor pressure (kPa); e_a_ means actual vapor pressure (kPa); e_s_ − e_a_ is the saturation vapor pressure deficit (kPa); ∆ means slope vapor pressure curve (kPa °C^−1^); γ is the psychrometric constant (kPa °C^−1^), and Kc means crop factors.
(3) The gray water used to assimilate nitrogen contamination from fertilizers is evaluated as gray water consumption. The WF_gray_ of growing a crop can be calculated as follows (Hoekstra et al. (2009) [31]):
  (7) $${\mathrm{WF}}_{\mathrm{gray}}=\frac{\left(a{\times \mathrm{AR})/(C}_{\max}-C_{\mathrm{nat}}\right)}{Y}$$
  where a is the leaching rate of N fertilizer (%); AR is the amount of N fertilizer used per unit area of a crop (kg hm^−1^); C_max_ means the maximum permissible concentration of nitrogen per unit volume of water (kg m^−3^), under current environmental water quality standards; C_nat_ represents the natural concentration of nitrogen per unit volume of water (kg m^−3^), and Y refers to the crop yield (t hm^−1^).

The leaching rate of N fertilizer is determined to be 10% according to the first China Pollution Source Census-Manual of Fertilizer Loss Coefficient for Agricultural Pollution Sources; the C_max_ value is determined to be 0.01 kg m^−3^, according to the Class III water standard in Environmental Quality Standards for Surface Water (GB 3838-2002); C_nat_ value is usually set to 0. Here, we ignored the effect of pesticides and other fertilizers due to lack of accessed data.

### 3.2. LMDI Factor Decomposition Model

The LMDI method was developed by Ang in 1998, and it has been widely used to analyze the driving forces of the changes in CO_2_ emissions or energy consumption [39,40]. Compared to other decomposition methods, the LMDI method has the advantages of expressing in a simple form with no residual errors and is especially suitable for models with time-series data, so has been recommended for general use [41].

Existing research on the decomposition of driving factors of water resources utilization by LMDI model, Zhang et al. (2015) [42] adopted LMDI model to decompose it into economic development level, population size, industrial water use efficiency and water resources development and utilization level, when studying the changes of total water consumption and water use efficiency in Urumqi from 1995 to 2012. Based on the improved Laspeyres model and LMDI model, Shang et al. (2016) [43] decomposed the driving forces of industrial water consumption into output force, technical force and structural force. Regarding the LMDI model combined with the theory of WF, when studying China’s provincial gray water footprint characteristic, Zhang et al. (2019) [44] decomposed the driving force of the gray WF into economic effects, strength effects, technology effect and development effect, based on the Kaya equation and LMDI model. Kang et al. (2017) [45] decomposed the driving forces of WF changes caused by food consumption into population, food consumption structure, food consumption level, water intensity and population ratio. Yang et al. (2014) [46] decomposed the driving factors of changes in the WF of food consumption into population, diet, and agricultural practices (unit water production). This paper draws on previous studies and selects the addition model to decompose the driving factors of WF changes in Gansu Province based on the difficulty of interpretation of the driving factors. The total agricultural WF consumption can be described as follows:

(8) $$\mathrm{WF}=\sum_{i}\frac{{\mathrm{WF}}_{i}}{\mathrm{WF}}\frac{\mathrm{WF}}{G}\frac{G}{P}P=\sum_{i}S_{i}\mathrm{TEP}$$

In Equation (8), WF denotes the total water resource consumption for agriculture (10^8^ m^3^); WF_i_ represents the WF of the ith group (10^8^ m^3^), i = 1, 2, denoting three major staple crops and major cash crops; G represents the agricultural GDP (100 million yuan); P refers to the number of permanent residents in rural areas (10 thousand people). S_i_ refers to the water footprint structure, which is the proportion of the water footprint of the ith group to the total agricultural water footprint consumption; T represents agricultural water efficiency, which is measured by the ratio of total agricultural water footprint consumption to agricultural GDP; E is the ratio of agricultural GDP to population size, representing agricultural economic activity. Assuming that the time changes from 0 to t, the amount of change in WF can be decomposed into four parts,

ΔWF = ΔWF_I_ + ΔWF_S_ + ΔWF_C_ + ΔWF_P_ (9)

Equation (9) is further sorted out as follows:

(10) $${\Delta \mathrm{WF}}_{I}=\frac{{\mathrm{WF}}_{t}-{\mathrm{WF}}_{t-1}}{{\mathrm{lnWF}}_{t}-{\mathrm{lnWF}}_{t-1}}\times \ln\left(\frac{I_{t}}{I_{t-1}}\right)$$

(11) $${\Delta \mathrm{WF}}_{S}=\frac{{\mathrm{WF}}_{t}-{\mathrm{WF}}_{t-1}}{{\mathrm{lnWF}}_{t}-{\mathrm{lnWF}}_{t-1}}\times \ln\left(\frac{S_{t}}{S_{t-1}}\right)$$

(12) $${\Delta \mathrm{WF}}_{C}=\frac{{\mathrm{WF}}_{t}-{\mathrm{WF}}_{t-1}}{{\mathrm{lnWF}}_{t}-{\mathrm{lnWF}}_{t-1}}\times \ln\left(\frac{C_{t}}{C_{t-1}}\right)$$

(13) $${\Delta \mathrm{WF}}_{P}=\frac{{\mathrm{WF}}_{t}-{\mathrm{WF}}_{t-1}}{{\mathrm{lnWF}}_{t}-{\mathrm{lnWF}}_{t-1}}\times \ln\left(\frac{P_{t}}{P_{t-1}}\right)$$

In the equation, ΔWF_I_, ΔWF_S_, ΔWF_C_ and ΔWF_P_ respectively represent the technical effect, structural effect, economic effect, and population effect, that is, the WF changes caused by changes in agricultural water efficiency, agricultural production structure adjustment, agricultural economic growth, and changes in agricultural population size.

### 3.3. Tapio Decoupling Index

The Tapio decoupling indicator reflects the relative change of resource environment with economic development in the ratio of growth rate, quantitatively describes the divergence of resource environment and economic development in the range of elastic values, breaks the time limit and overcomes the drawback of OECD indicator that there is not a strong stability. Therefore, this paper draws on the Tapio decoupling index to mark the decoupling index between the WF change in Gansu Province and agricultural economic growth as the decoupling indicator for agricultural development.

(14) $$D(\mathrm{WF},G)=\frac{{\Delta \mathrm{WF}/\mathrm{WF}}_{t-1}}{{\Delta G/G}_{t-1}}$$

In the equation, D(WF,G) is the decoupling elasticity between the gross agricultural product value G and the water footprint WF in Gansu Province, ΔWF is the variation in the WF of the current period relative to the previous period WF_t−1_, and ΔG is the variation in the current period compared with the total agricultural production value G_t−1_ in the previous period. Tapio model decoupling status is divided into decoupling, connection and negative decoupling three situations [22], the specific classification is shown in Table 1.

According to Equations (10)–(14), combined with the LMDI index decomposition model, a WF decoupling index decomposition model can be derived as follows,

(15) $$D(\mathrm{WF},G)=\frac{{\Delta \mathrm{WF}/\mathrm{WF}}_{t-1}}{{\Delta G/G}_{t-1}}=\frac{{(\Delta \mathrm{WF}}_{I}{+\Delta \mathrm{WF}}_{S}{+\Delta \mathrm{WF}}_{C}{+\Delta \mathrm{WF}}_{P}{)/\mathrm{WF}}_{t-1}}{{\Delta G/G}_{t-1}}$$

D(WF, G) = D_I_ + D_S_ + D_C_ + D_P_ (16)

In Equation (16), D_I_, D_S_, D_C_, D_P_ are the changes in the decoupling factors caused by technological effects, structural effects, economic effects and population effects. The decoupling results can be calculated and collated according to the corresponding relationship between the decoupling index and decoupling status in Table 1.

### 3.4. Data Source and Description

The research period of this paper is from 2006 to 2015. The research objects include the three major staple crops of rice, corn and wheat and the three major cash crops of cotton, soybean and potato. The crop water requirements and crop production data required for agricultural WF assessment are mainly come from the Food and Agriculture Organization of the United Nations (FAO) and the National Meteorological Information Center. The irrigation quota was based on Municipal Water Quota of Main Sectors in Gansu Province. Gansu Statistical Yearbooks supplied data related to gross value of agricultural production, agricultural added value, population, crop yield, and fertilization.

## 4. Empirical Study

### 4.1. Descriptive Statistical Analysis

This paper calculates the WF of the three major staple crops and major cash crops in Gansu Province from 2006 to 2015 by CROPWAT8.0 model. The results are shown in Figure 1.

#### 4.1.1. WF of Agriculture in Gansu Province

**Figure 3:**
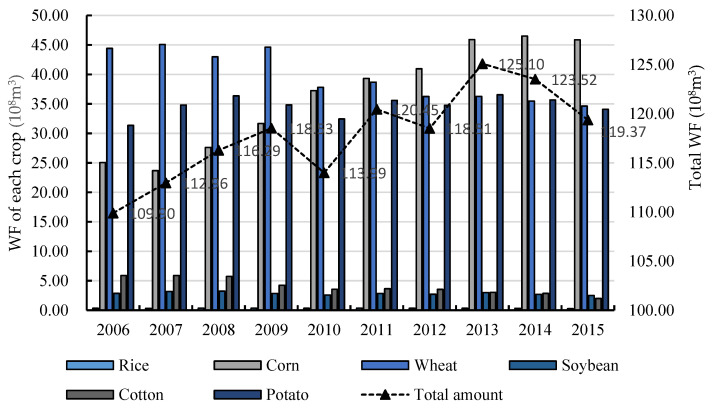
WF of crops in Gansu Province from 2006 to 2015.

It can be seen from Figure 3 that Gansu’s agricultural WF reached 109.90 × 10^8^ m^3^, 113.99 × 10^8^ m^3^ and 119.37 × 10^8^ m^3^ in 2006, 2010, and 2015, respectively. The average annual WF was 117.86 × 10^8^ m^3^, experiencing the process of rising first and then volatility falling. This trend was consistent with the result of Xu et al.’s research (2015) [26], who calculated the WF of crop production in Beijing from 1978 to 2012. In detail, the agricultural WF in Gansu Province increased steadily from 109.10 × 10^8^ m^3^ in 2006 to 118.53 × 10^8^ m^3^ in 2009 and declined in 2010 and 2012. In 2013, the agricultural WF in Gansu Province reached the highest value of 125.10 × 10^8^ m^3^, and it has continuously dropped to 119.37 × 10^8^ m^3^ in the following two years. From the perspective of crop structure, the WF of wheat, corn and potato products in Gansu during the period of investigation was relatively high, respectively reaching 39.62 × 10^8^ m^3^, 36.38 × 10^8^ m^3^ and 34.64 × 10^8^ m^3^, followed by cotton (4.04 × 10^8^ m^3^) and soybean (2.84 × 10^8^ m^3^) and rice products (0.34 × 10^8^ m^3^); the WF of the products differs greatly, which is highly related to the local climate and crop yields.

We could find that agricultural WF in Gansu Province was significantly related to crop types, and there were significant heterogeneity characteristics of WF among different crops. Based on the calculation results of agricultural WF, it could be seen that the overall agricultural WF in this area showed an upward trend of fluctuation, and the WF of wheat, corn and potato was relatively high, while the WF of cotton, soybean and rice products was less than one-tenth of the former. The large difference in WF of different crops indicates that the agricultural sector in Gansu Province needs to pay attention to the local planting structure and take rationalized planting measures that suit the local environment.

#### 4.1.2. Relationship between Agricultural Economic Growth and WF Changes

**Figure 4:**
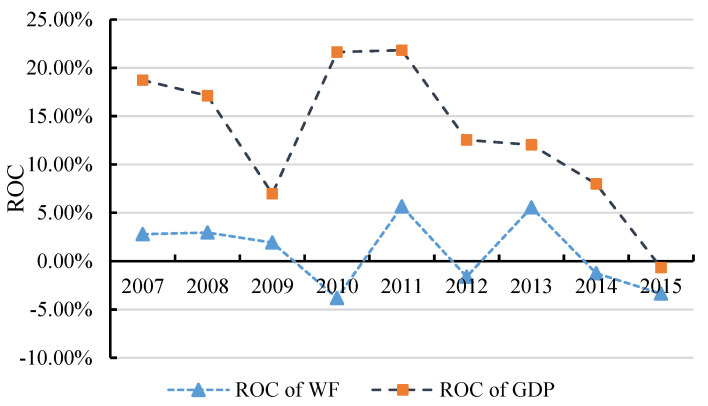
Gansu’s agricultural WF and economic growth rate of change (ROC) line chart.

Figure 4 shows the change of agricultural WF and the growth trend of agricultural GDP in Gansu Province from 2006 to 2015. By observing the changes in agricultural output in Gansu Province, it can be seen that from 2007 to 2009, the agricultural economic growth rate of Gansu Province has decreased greatly year by year. From 2009 to 2010, the economic growth rate rose sharply, then it fluctuated and declined with each passing year, and the growth rate of agricultural output gradually slowed down. During the research period, in terms of the rate of change (ROC), the WF of Gansu’s agricultural sector decreased with the decrease of agricultural GDP in Gansu Province in five years (2009, 2011, 2012, 2014 and 2015). The change of agricultural WF in the sample period showed a downward trend of fluctuations year by year, and the effect of matching the agricultural economic growth rate was obvious. In general, the annual trends of both were concordant and the agricultural economic growth rate was always higher than the WF growth rate, indicating that there is a high correlation between changes in agricultural WF and agricultural economic growth in Gansu Province.

It should be specially explained that the technological effects, structural effects, economic effects and population effects have an entirely different influence on the changes of agricultural WF, and the ROC of agricultural WF is jointly decided by these 4 factors. Figure 4 shows that the ROC of agricultural WF fluctuated significantly from 2010 to 2015. The reason was that the stimulative effect of economy and structure was offset by the inhibition effect of technology and population, and the more obvious side could determine the sign of the final ROC of WF. However, why does the economic factor have a stimulative effect, while the population factor has an inhibition effect? This should be judged by the trends shown in the raw data. Through observing the changes in the raw data, we could find that agricultural GDP kept increasing during the research period in Gansu Province, so it needed more water to maintain the economic development. Otherwise, the amount of agricultural population kept decreasing, which meant that much less water could satisfy the needs of local people.

### 4.2. Analysis of Driving Effects of Agricultural WF Changes

According to Equations (9)–(13), in the LMDI decomposition model, the driving factors of WF change in Gansu Province are quantified in four aspects, technical effect, structural effect, economic effect and population effect, and explore the driving effects and leading factors of agricultural WF changes in Gansu Province from 2006 to 2015 and in the 11th and 12th Five-Year Plan periods. The results are shown in Table 2.

In general, the agricultural WF in Gansu Province increased by 113.27 × 10^8^ m^3^ from 2006 to 2015, and the total effect was positive. In detail, the technological effect has made a contribution of 41.35% to inhibit the increase of WF, and the cumulative decrease of agricultural WF in the sample period has fallen by 643.24 × 10^8^ m^3^. On the contrary, the economic effect is the leading factor driving the increase of agricultural WF. An increase of 782.76 × 10^8^ m^3^ contributes 50.31% to the change of WF. From the angle of time, agricultural economic growth and agricultural production technology improvement have always been the main driving factors and main inhibitors of the driving effect of agricultural WF changes in Gansu Province. However, the changes in the value of both are unstable, and structural effect and population effect contribute less to the changes in local agricultural WF. During the 11th Five-Year Plan period, the total effect of agricultural WF changes in Gansu Province was positive, with a cumulative increase of 10.55 × 10^8^ m^3^. In detail, the improvement of agricultural technology caused the reduction of agricultural WF by 78.48 × 10^8^ m^3^, making a 41.94% contribution and occupying a dominant position. The total effect of agricultural WF changes in Gansu Province during the 12th Five-Year period was still not positive, but compared with the 11th Five-Year period, the amount of WF changes has decreased by 5.18 × 10^8^ m^3^. At the same time, the dominant factor has changed from technological effect to economic effect, showing that the improvement of agricultural planting technology has gradually reduced the impact of agricultural WF changes.

Compared to the result of Zhao et al.’s research (2014) [25], we could find that the technological factor always is the main factor that has an inhibition effect on the changes of the agricultural WF, and the economic factor is the largest driver of the growth of WF. Essentially, technological effect means the improvement in water use efficiency, which should be attributed to the wide application of water-saving technologies and the effort of local government to set some corresponding agricultural policies. On the other hand, although China’s rapid economic development is mainly driven by urbanization, many farmers in remote areas are still highly reliant on crop planting to make a living. Due to the strong desire of many farmers to get rid of poverty, the agricultural WF has a significant increase accordingly.

### 4.3. The Decoupling Relationship between Agricultural WF Changes and Agricultural Economic Growth

The Tapio decoupling index was used to measure the decoupling relationship between changes in agricultural WF and agricultural economic growth in Gansu Province from 2006 to 2015. Table 3 shows the results of the decoupling calculation between water footprint WF and gross agricultural product G in Gansu Province.

#### 4.3.1. The Decoupling Status of Changes in Agricultural WF and Economic Growth

It can be seen from Table 3 that the agricultural WF in Gansu Province exhibited strong decoupling with the agricultural economic growth for four years (2009, 2011, 2013 and 2014) and weak decoupling for five years (2006, 2007, 2008, 2010 and 2012). Overall, they have undergone a process from “weak” to “strong”, and the decoupling status is generally considered satisfactory. The results differ from that of Zhang et al.’s research (2014) [12], in which the trend of strong decoupling occurred for four years, weak decoupling and recessive decoupling occurred for one year each, and the volumes of agricultural WF did not increase in proportion with crop yield in the Heilongjiang land reclamation area (HLRA) in four years. In particular, Gansu’s agricultural economic growth and WF were weakly decoupled from 2006 to 2008. During this time, agricultural WF increased while experiencing economic growth, and the latter’s growth rate was smaller than the former’s. From 2009 to 2013, its decoupling status showed a “strong decoupling–weak decoupling” alternating state with large fluctuations, because Gansu’s planting underwent structural adjustment and transformation. From 2013 to 2015, Gansu’s agricultural WF and agricultural economic growth reached a strong decoupling status, indicating that agricultural output increased while agricultural WF decreased, achieving the best decoupling status. This result has to do with the overall guiding ideology of “water-saving and efficiency-enhancing and high-efficiency water-saving irrigation development” advocated and implemented by local government, which is very effective in suppressing agricultural water consumption. Moreover, we can greatly benefit from the deepening of the application of water-saving technologies. As we know, it is of great importance to establish an environmentally friendly and resource-saving agricultural production system for the development of China’s agricultural sector. In the future, we should insist on this production system and keep the stability of the current decoupling status.

#### 4.3.2. Analysis of Changes in Decoupling Factors of Agricultural WF

**Figure 5:**
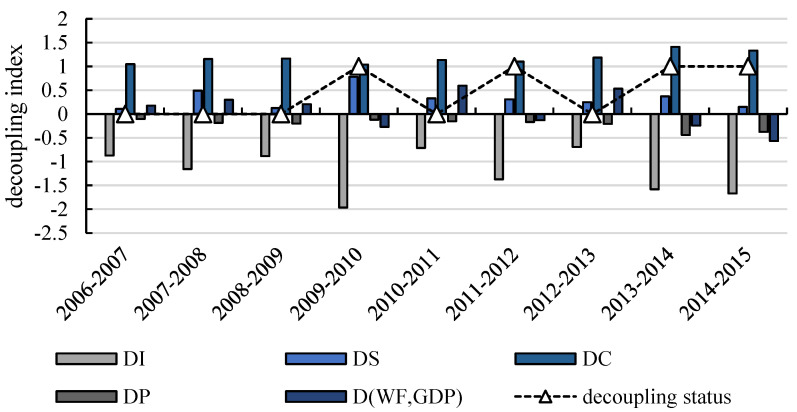
The decomposition results of the decoupling index of Gansu’s Province agricultural WF from 2006 to 2015. Note: “0” in the decoupling status means weak decoupling, “1” means strong decoupling.

Based on the previous analysis, combined with the LMDI index decomposition model, the WF decoupling index of Gansu Province is decomposed to explore the main driving factors affecting the change of agricultural WF, and the changes in agricultural WF decoupling factor in Gansu Province at various stages are shown in Figure 5.

As shown in Figure 5, economic factors and structural factors were the driving factors that hindered the decoupling of agricultural WF in Gansu Province during the research period, and the influence of structural factors and population factors on the decoupling of agricultural WF in Gansu Province was limited. This result is consistent with that shown in Table 2 and Table 3. The promotion effect of technological factors on decoupling was greater than the inhibition effect of income factors, occupying a dominant position, and the overall decoupling effect was satisfactory. In terms of specific time periods, the inhibition effect of economic factors on the decoupling of agricultural WF in Gansu Province was relatively stable. The annual average decoupling index was 1.17, making a contribution of approximately 41.67%, and the technological factors annual decoupling index was −1.21, making a contribution of about 40.33%. On the whole, it is slightly lower than the inhibition effect of economic factors. In detail, the contribution rate of technological factors to decoupling was as high as 50.34% from 2009 to 2010 but fell to 29.72% from 2012 to 2013, indicating that the interannual change of technological factors in promoting the decoupling of agricultural WF in Gansu Province is relatively large.

## 5. Conclusions

From the perspective of WF, this paper establishes a decoupling model of agricultural economic growth and agricultural WF changes and explores the changes of the driving factors of WF using the LMDI decomposition method, based on the panel data of Gansu Province from 2006 to 2015.

The main findings of our paper are as follows. The agricultural WF in Gansu Province was significantly related to crop types, and there are significant heterogeneity characteristics of WF among different crops. The overall agricultural WF showed an upward trend of fluctuation, and the WF of wheat, corn and potato was relatively high. In terms of driving factors for the change of agricultural WF, economic effects were the main factors that promoted the growth of WF, and the improvement of agricultural production technology was the main inhibition factor. On the whole, the promotion effect brought by economic growth is greater than the inhibition effect of technological improvement. Compared with the 11th Five-Year Plan period, the total effect of Gansu’s agricultural WF changes during the 12th Five-Year Plan period was still positive, but the main driving factor changed from technological effect to economic effect. Moreover, the decoupling of agricultural WF and agricultural economic growth in Gansu province mainly experienced two stages of weak decoupling and strong decoupling, and the decoupling status was relatively satisfactory. To be specific, structural factors and population factors have little effect on decoupling, and economic factors have a relatively stable inhibition effect, and technological factors effect a large interannual change in the driving effect of decoupling, which was slightly lower than the inhibition effect of economic factors.

Based on previous analysis, there are several strategies available to reduce the current impacts of agriculture production on water use and keep a satisfactory decoupling status. Firstly, the large difference in WF of different crops indicates that the agricultural sector in Gansu Province should attach more importance to local planting structure, adjust planting structure according to local conditions and improve the structure effects on the changes of agricultural WF. Secondly, as the inhibition effect of technological factors is gradually becoming weak, we should put more emphasis on efforts to improve water use efficiency. The government should further increase the investment in construction of agricultural water-conserving infrastructure such as plastic mulches, sprinkler irrigation systems and drip irrigation systems. Moreover, it is of great importance to promote the widespread adoption of water-saving technologies in China’s agricultural production system and, especially, encourage farmers to adopt it. Thirdly, it is necessary to adhere to the priority of saving water and further explore the driving factors of the growth of agricultural WF. In the past decades, the pattern of economic development and the structure of food consumption has changed greatly. Under such a background, government should set scientific agricultural policies and adopt appropriate irrigation management practices to realize sustainable development.

In terms of the contributions of this paper, the first is that based on the assessment and evaluation of water footprint, a water footprint method is introduced into the decoupling analysis to study the decoupling status between water use and economic development. The second is that, focusing on the agricultural sector that consumes the most water, this paper adopts the LMDI method to decompose the decoupling index of agricultural water footprint and explore the driving factors for the decoupling of agricultural water footprint. All in all, the analytical method can be adopted by other researchers to discuss the degree of divergence between economic development and water footprint in other sectors, not only the agricultural sector.

One limitation of the study regarding research object is that we calculated the WF of the most representative crops, including 3 staple crops and 3 cash crops, not being able to cover all crops involved in the agricultural production in Gansu Province. Besides, the results from sub-regions in Gansu Province are not available. Furthermore, we ignore the effect of pesticides and other fertilizers due to lack of access to data. Another limitation of the study regarding gray WF computation is that we use the data according to the first China Pollution Source Census-Manual of Fertilizer Loss Coefficient for Agricultural Pollution Sources and the Class III water standard in Environmental Quality Standards for Surface Water (GB 3838-2002), and the leaching rate of N fertilizer and C_max_ value data do not vary by crop. Moreover, we use the data of the irrigation parameter of each crop that comes with the CROPWAT8.0 software, and we do not set this value. In future research, we will go to Gansu Province or other study areas to conduct a field survey so that we are able to collect more accurate data of some main crops and measure their specific effects on the changes of the agricultural WF and decoupling status.

## Figures and Tables

**Table 1:** The division of decoupling indicators.

Decoupling		ΔGDP	ΔWF	D(WF,GDP)	Status Description
Type	Status				
Coupling	expansive	>0	>0	0.8 ≤ D ≤ 1.2	Both increase, relatively synchronized
	recessive	<0	<0	0.8 ≤ D ≤ 1.2	Both decrease, relatively synchronized
Decoupling	weak	>0	>0	0 ≤ D < 0.8	Both increase, GDP changes faster
	strong	>0	<0	D < 0	GDP increases, WF decreases, best status
	recessive	<0	<0	D > 1.2	Both decrease, WF changes faster
Negative decoupling	expansive	>0	>0	D > 1.2	Both increase, WF changes faster
	strong	<0	>0	D < 0	WF increases, GDP decreases, worst status
	weak	<0	<0	0 ≤ D < 0.8	Both decrease, GDP changes faster

**Table 2:** Driving effect of agricultural WF in Gansu Province from 2006 to 2015.

Year	ΔWF_I_	ΔWF_S_	ΔWF_C_	ΔWF_P_	Total Effect	Principal Factor	
						Stimulative	Withholder
2006–2007	−15.22	1.85	18.29	−1.86	3.06	economic *	technological
2007–2008	−12.82	5.42	12.8	−2.08	3.33	economic	technological *
2008–2009	−9.56	1.37	12.6	−2.17	2.24	economic *	technological
2009–2010	−33.15	13.16	17.51	−2.04	−4.53	economic	technological *
2010–2011	−7.72	3.55	12.28	−1.66	6.45	economic *	technological
2011–2012	−20.28	4.53	16.29	−2.47	−1.93	economic	technological *
2012–2013	−8.6	3.07	14.68	−2.57	6.58	economic *	technological
2013–2014	−10.21	2.39	9.09	−2.85	−1.58	economic	technological *
2014–2015	−12.26	1.1	9.77	−2.76	−4.15	economic	technological *
2006–2015	−643.24	51.74	782.76	−77.99	113.27	economic *	technological
11th five-year	−78.48	25.35	73.48	−9.8	10.55	economic	technological *
12th five-year	−59.07	14.64	62.12	−12.31	5.37	economic *	technological

* Represents the dominant factor.

**Table 3:** Decoupling status of agricultural WF and economic growth in Gansu Province from 2006 to 2015.

Year	D_I_	D_S_	D_C_	D_P_	D(WF,GDP)	Status
2006–2007	−0.87	0.11	1.05	−0.11	0.18	weak
2007–2008	−1.16	0.49	1.16	−0.19	0.30	weak
2008–2009	−0.88	0.13	1.17	−0.20	0.21	weak
2009–2010	−1.97	0.78	1.04	−0.12	−0.27	strong
2010–2011	−0.71	0.33	1.14	−0.15	0.60	weak
2011–2012	−1.37	0.31	1.10	−0.17	−0.13	strong
2012–2013	−0.69	0.25	1.18	−0.21	0.53	weak
2013–2014	−1.58	0.37	1.41	−0.44	−0.24	strong
2014–2015	−1.67	0.15	1.33	−0.38	−0.57	strong
